# Strength Training Rehabilitation Incorporating Power Exercises (STRIPE) for individuals with patellofemoral pain: a randomised controlled trial protocol

**DOI:** 10.1136/bmjsem-2022-001482

**Published:** 2023-01-17

**Authors:** Neal R Glaviano, L Colby Mangum, David M Bazett-Jones, Lindsay J DiStefano, Michael D Toland, Michelle Boling

**Affiliations:** 1 Department of Kinesiology, University of Connecticut, Storrs, Connecticut, USA; 2 Institute of Exercise Physiology and Rehabilitation Science, University of Central Florida, Orlando, Florida, USA; 3 Department of Exercise & Rehabilitation Sciences, The University of Toledo, Toledo, Ohio, USA; 4 The Herb Innovation Center, Judith Herb College of Education, University of Toledo, Toledo, Ohio, USA; 5 Department of Clinical & Applied Movement Sciences, University of North Florida, Jacksonville, Florida, USA

**Keywords:** Hip, Knee, Randomised controlled trial, Rehabilitation, Sports rehabilitation programs

## Abstract

Patellofemoral pain (PFP) is a chronic condition that presents with patellar pain during various daily and recreational activities. Individuals with PFP have a wide range of impairments that result in long-term disability and reduced quality of life. Current interventions target hip muscle weakness with strength-based exercises, but recurrence rates are as high as 90%. A single feasibility study demonstrated success with power-based exercises; however, there is limited evidence evaluating pain or self-reported function in larger cohorts, and no study has assessed recurrence rates. This protocol details a study evaluating a strength-based rehabilitation programme compared with a strength-based programme incorporating power-based exercises in individuals with PFP. This single-blinded randomised controlled trial will evaluate 88 participants with PFP, aged 18–40 years old. Participants will be recruited from three universities, the surrounding community and sports medicine clinics. Participants will receive three telemedicine rehabilitation sessions a week for 6 weeks. The rehabilitation programme will consist of either strength-based exercises or a combination of power and strength-based exercises. Pain, subjective function and recurrence rates will be assessed at baseline, immediately after the intervention and at four follow-up time points: 6-month, 12-month, 18-month and 24-month postintervention. We will also assess neuromuscular function of the hips and global rating of change at each postintervention time point. Trial registration number NCT05403944.

**WHAT IS ALREADY KNOWN ON THIS TOPIC**

- Clinicians commonly prescribe strength-based rehabilitation programmes to treat patellofemoral pain; however, many patients do not demonstrate improvements in strength.
- Individuals with patellofemoral pain are not satisfied with their knee function within months after completing rehabilitation and report disability for years after treatment.

**WHAT THIS STUDY ADDS**

- Provides information if incorporating power-based exercises compared with strength-based exercises are more effective at improving pain, self-reported function and neuromuscular function of the hip muscles in individuals with patellofemoral pain.
- Provides evidence if a rehabilitation programme with power-based exercises reduces the recurrence rates of patellofemoral pain for 2 years after treatment.

**HOW THIS STUDY MIGHT AFFECT RESEARCH, PRACTICE OR POLICY**

- This study would be a paradigm shift in the type of exercises prescribed to individuals with patellofemoral pain, as power-based exercises are limited in the literature and clinical practice.

## Introduction

Patellofemoral pain (PFP) is one of the most common knee pathologies in the general population and military personnel.^1^ The incidence of PFP in the military ranges from 9.7 to 571.4 per 1000 person-years, with females at 2.2 times greater risk than males.^1 2^ The annual prevalence rate in the general adult population is 22.7%.^1^ PFP accounts for 7% of all diagnoses in patients who seek medical care^3^ and up to 25% of all treatment for knee-related injuries within sports medicine clinics.^4^ Persistent knee pain, disability and impaired joint-related and health-related quality of life are common in individuals with PFP.^5 6^

The aetiology of PFP is unknown. Increased stress on the patellofemoral joint due to altered lower extremity mechanics is proposed to be the primary mechanism of PFP.^7^ Altered movement patterns are common in individuals with PFP during tasks, such as single-leg squat, stair ambulation and running,^8 9^ and are associated with greater knee pain and worse subjective function.^10^ Hip musculature is a major influence on the control of these lower extremity mechanics, and adults with PFP are 20%–28% weaker in hip strength when compared with asymptomatic adults.^11 12^

Clinicians prescribe strengthening exercises focused on weak gluteal muscles,^13 14^ which produces positive short-term outcomes but suboptimal long-term outcomes. Over 90% of patients with PFP report knee pain and symptoms greater than 15 years following initial diagnosis.^6^ While most patients with PFP seek medical care,^8^ over 60% are not satisfied with their knee function within 3 months of concluding treatment.^15^ Numerous studies targeting hip abductor strength produce minimal improvements,^16^ and hip strength gains are lost within months of concluding treatment.^16 17^

The lack of strength changes after hip-focused strengthening interventions suggests that there may be additional meaningful factors of neuromuscular function in adults with PFP. Rate of torque development (RTD) quantifies one’s ability to develop force rapidly and is sensitive at detecting chronic neuromuscular dysfunction.^18^ Adults with PFP have significant gluteal RTD deficits (55%) compared with asymptomatic adults, which represents almost twice the muscle function deficit when compared with strength measures (20%–28%).^19 20^ Not only does RTD better identify deficits in hip muscle function, RTD also more closely resembles demands of various functional tasks compared with isolated hip strength.^21^ RTD is an important measure of neuromuscular function in individuals with PFP that clinicians need to target during treatment.

Since gluteal RTD deficits are greater than strength deficits, interventions need to be developed specific to those deficits. Power-based exercises, which require a heavy resistance being conducted at a high peak force, are one intervention that has supportive evidence to improve RTD.^18^ Power-based exercises are more effective at enhancing RTD than strength-based exercises in adolescents, elderly and various pathological populations.^22 23^ To date, only a single feasibility study has evaluated the effect of power-based exercises on individuals with PFP.^24^ Power-based exercises targeting hip muscles successfully improved self-reported function, reduced pain and improved hip abduction and extension strength and power.^24^ Yet, this study only included 10 participants, did not include a strength-training comparison group and reported low adherence (33%) during weeks 7–12 of the intervention.^24^ Low adherence in late stages reduces the clinical applicability of the findings and negatively impacts the understanding of adequate dosage of power-based exercises for the treatment of PFP.^24^ Therefore, it is essential to conduct a randomised controlled trial (RCT) with a larger sample to overcome the limitations that reduce the clinical applicability.

### Aims and objectives

The proposed study will compare a strength-based rehabilitation programme, the standard of care (SOC), to strength training rehabilitation incorporating power exercises (STRIPE). The exercises in both groups will target the hip abductors, hip extensors, core and quadriceps muscles. Our primary outcomes are self-reported knee pain measured by the Visual Analogue Scale (VAS), self-reported function measured by the Anterior Knee Pain Scale (AKPS) and recurrence rates. Recent evidence suggests that two in every three individuals with PFP report symptoms up to 2 years after seeking care^25^; therefore, we will assess PFP recurrence rates for 2 years. The proposed interventions target gluteal muscle function, which will be collected as secondary outcome variables (hip RTD and frontal plane kinematics).

### Study hypothesis

The study hypothesis is that participants assigned to the STRIPE group will have a greater decrease in pain, greater improvements in self-reported function and lower PFP recurrence rates compared with those in the SOC group. Additionally, participants in the STRIPE group will have greater improvement in hip RTD and frontal plane kinematics compared with SOC.

## Methods

### Study design

This is a single-blinded RCT with two interventions, SOC and STRIPE. We adhered to the Standard Protocol Items: Recommendations for Intervention Trials checklist for this report (online supplemental file 1).^26^

10.1136/bmjsem-2022-001482.supp1 Supplementary data

### Patient and public involvement

There was no formal involvement for patients or public individuals in the development of this study design.

### Setting

We will conduct this trial in three laboratories, the Sports Optimisation and Rehabilitation Laboratory at the University of Connecticut, Storrs, Connecticut, USA; the Rehabilitation, Athletic assessment & Dynamic Imaging Laboratory at the University of Central Florida, Orlando, Florida, USA; and the Motion Analysis & Integrative Neurophysiology Laboratory at the University of Toledo, Toledo, Ohio, USA.

### Eligibility and screening

Participants will be sought through flyers, social media posts, sports medicine physician referrals, and participation in previous PFP studies. Participants will be 18–40 years old and screened for eligibility with the inclusion/exclusion criteria defined by the Patellofemoral Pain Consensus statement^27^ (box 1). The intervention will be delivered through telemedicine, requiring participants to have a computer with internet access and camera/audio capabilities. Each site’s principal investigator (site-PI) will screen participants to ensure eligibility and consent participants. The three investigators at each site will complete an eligibility training and screening procedures session before participant enrolment.

Box 1 **Inclusion and exclusion criteria**

Inclusion criteria

- Insidious onset of peripatellar or retropatellar pain greater than 3 months.
- Worst pain in the previous month of 3/10 with two of the following tasks:
  - Prolonged sitting.
  - Jumping.
  - Squatting.
  - Kneeling.
  - Running.
  - Stair ambulation.

Exclusion criteria

- Other forms of anterior knee pain
  - Osgood-Schlatter.
  - Tendon pain.
  - Bursitis.
- History of lower extremity surgery.
- History of patella subluxation.
- Meniscal injury, assessed by clustered special tests: history of ‘catching’ or ‘clocking’, pain with forced hyperextension, pain with maximal knee flexion, pain with McMurray test and joint line tenderness.
- Ligamentous instability, assessed by the anterior drawer, Lachman, posterior drawer, varus/valgus stress tests.
- Referred pain from the lumbar spine.
- Mental capacity for human subjects to consent to participating in the study.

### Randomisation, blinding and treatment allocation

Participants will be assigned to a treatment group by block randomisation, with a computer-generated online research randomiser (http://www.randomizer.org), completed by the statistician (MDT) prior to study enrolment. We will use a four-block randomisation approach for each clinical site, to ensure equal groups across sites. The University of Connecticut site will enroll 44 participants, resulting in 11 equal blocks of randomisation. The University of Central Florida and University of Toledo will each collect 22 participants, resulting in five 4 blocks of randomisation and a final block of 2 participants to allow for equal randomisation. An additional four-block randomisation was provided for each site in the instance a participant was assigned to a group but dropped out before any study data were collected. Group assignment will be maintained with a master list and allocation concealed from the site-PIs.

The proposed study will include blinding the research members conducting the baseline and postintervention assessments from group allocation, while separate team members will perform all treatments and will not conduct baseline or postintervention assessments. The participants will not be blinded, as reviewing the informed consent will outline group differences. To control for selection bias, the randomisation will be completed by the statistician who will not be directly associated with data collection or intervention. Additionally, the statistician will oversee randomisation with concealed allocation in opaque envelopes that will only be opened after baseline testing. Participants will be assigned a random participant number per site, maintained in an encrypted and secure document by site-PIs, ensuring confidentiality. We will also collect numerous secondary variables, such as psychological factors, coping strategies, physical activity and lifestyle factors, which we aim to reduce bias by using them as covariates if they influence the primary outcomes.

### Intervention

The two interventions, SOC and STRIPE, were developed with the Template for Intervention Description and Replication checklist for telehealth,^28^ Consensus on Exercise Reporting Template for exercise interventions^29^ and Toigo and Boutellier for resistance training interventions (online supplemental file 2–4).^30^

The intervention was modified from an existing protocol,^24^ and will consist of a 6-week intervention, totalling 18 rehabilitation sessions. Both groups will complete 3 weekly telemedicine sessions, each lasting 45–60 min. All participants will complete standardised exercises that include four components—hip extensor, hip abductor, core and quadriceps muscle groups. The SOC group will complete a strength training rehabilitation programme, whereas the STRIPE group will complete a strength training rehabilitation programme incorporating power-based exercises. Each week, SOC participants will complete three strength training sessions and STRIPE participants will complete two power and one strength training session. All participants will be provided a digital exercise pamphlet, including images with instructions, access to online videos of each exercise and general PFP educational information. Participants will be advised to avoid additional rehabilitation during the 6-week rehabilitation programme.

Strength and power-based rehabilitation sessions will adhere to guidelines from the American College of Sports Medicine (table 1).^31^ Starting load for both groups will be determined after the baseline session, to ensure each participant starts with the appropriate load. Participants will be provided varying levels of resistance bands at the initial visit to use throughout the intervention. Resistance will be monitored during the intervention to ensure exercises are challenging, but not resulting in volitional muscular failure. Hip abductor, hip extensor and core exercises will be initiated during week one and continued for the 6-week intervention, while quadriceps exercises will be introduced in the third week for both STRIPE and SOC groups.^24^

**Table 1:** Descriptors of power-based and strength-based rehabilitation sessions

	Power-based exercises	Strength-based exercises
Load magnitude	60% 1RM	60%–70% 1RM
Sets and repetitions	4×6	3×12
Time under tension	<1 s concentric and 1 s eccentric	2 s concentric and 2 s eccentric
Rest between sets	3–5 min	2–3 min
Rest between repetitions	<1 s	<1 s
Rest between sessions	Minimum of 48 hours	Minimum of 24 hours

The intervention sessions will be administered through supervised telemedicine to expand access for participants. The telemedicine sessions will allow the research team to facilitate supervision, progress exercises, individualise the exercise components and assess adherence of all sessions. The research team will provide individualised care from a private laboratory space and participants will complete sessions at their location of choice. All sessions will be delivered individually, and each session will be scheduled between the research team and participant. Telemedicine sessions will be conducted on their university sponsored platform (WebEx, Zoom, Teams) and will use password protection and waiting rooms to protect participant privacy. We will not record or store any electronic protected health information during the sessions to protect HIPAA. The research team administering the intervention are licensed athletic trainers or credentialed physical therapists with multiple years of clinical experience treating musculoskeletal conditions, including PFP. All research team members administering the intervention will complete training by the PI prior to enrolment. Training will include exercise descriptors, exercise feedback in a telemedicine format, progression/regression instructions, recording adherences and monitoring adverse events. The PI will complete fidelity assessments on the training topics every 3 months. Research team members will provide verbal encouragement to participants, monitor adherence and record session data (sets, repetitions, exercises, resistance, pain and exertion).

Exercise progression or regression will be individualised to the participant’s daily pain level and perceived effort with the modified rate of perceived exertion (RPE) scale (0—very light activity to 10—maximal effort). The target goal is pain less than 3/10 on the VAS and participants reporting between 7 and 9 on the RPE scale. Participants who report pain greater than 3/10 on a specific exercise will regress by reducing load or the exercise. Participants who report pain less than 3/10 and less than 7 on the RPE scale will progress the exercise by increasing resistance or advancing the task. No additional criteria for discontinuation or modifying the intervention will be administered; however, participants may elect to withdraw from the study at any time and the reason for withdrawal will be requested.

### Demographics and outcome measures

Demographic variables (height, mass, age, sex, ethnicity, symptom duration, unilateral/bilateral symptoms) will be recorded at baseline. We will collect primary and secondary outcomes adhering to the study timeline (table 2). Participants will also complete a health history questionnaire, which includes questions related to previous treatment, pain location, aggravating factors, crepitus and pain quality. Baseline data, demographic variables and outcome measures will adhere to the REPORT-PFP checklist (Online supplemental file). We will instruct participants not to take pain medication for 48 hours prior to data collection of a primary outcome.^32^

**Table 2:** Data collection timeline

		Baseline	Postintervention	6 months	12 months	18 months	24 months
Primary	Visual Analogue Scale	X	X	X	X	X	X
	Anterior Knee Pain Scale	X	X	X	X	X	X
	PFP recurrence			X	X	X	X
Secondary	Hip Rate of Torque Development	X	X				
	Fontal Plane Single Leg Squat Kinematics	X	X	X	X	X	X
Descriptive variables	PROMIS-10	X	X	X	X	X	X
	KOOS w/ PF-subscale	X	X	X	X	X	X
	Fear Avoidance Belief Questionnaire	X	X	X	X	X	X
	Pain Self-Efficacy Questionnaire	X	X	X	X	X	X
	International Physical Activity Questionnaire	X	X	X	X	X	X
	Global Rating of Change		X	X	X	X	X

KOOS, Knee injury and Osteoarthritis Outcome Score; PFP, patellofemoral pain; PROMIS-10, Patient-Reported Outcomes Measurement Information System-10.

### Primary outcomes

Pain will be measured with a VAS, quantifying the participant’s worst knee pain in the previous week. Pain will be assessed using a 10 cm line, with ‘no pain’ and ‘worst pain imaginable’ anchored on the left and right sides of the VAS, respectively. The distance between the left anchor and the participant denoted location will be measured as the worst pain score in the previous week. A change of 2 cm on a 10 cm VAS reflects the minimal clinically important difference in pain following treatment for patients with PFP.^33^ The VAS has fair to good test–retest reliability (Intraclass Correlation Coefficient (ICC)_(3,1)_ =0.56−0.83)) (1 week) and moderate concurrent validity (r=0.62 with AKPS and r=0.74 with Functional Index Questionnaire) in individuals with PFP.^33^ We will collect AKPS at baseline and all postintervention time points (table 2).

The AKPS is a 13-item questionnaire to assess self-reported knee function in patients with PFP. The AKPS ranges from 0 to 100 points, with lower scores representing greater knee disability. A 10-point change on the AKPS reflects the minimal clinically important difference in self-reported function following treatment for patients with PFP.^33^ The AKPS has good test-retest reliability (ICC _(3,1)_ =0.81) and moderate concurrent validity (r=0.70) with usual pain in the PFP population, and significant responsiveness.^33^ AKPS will be collected at baseline and all postintervention time points (table 2).

PFP recurrence will adhere to previously established methods^17^ and be assessed at four time points: 6, 12, 18 and 24 months following the intervention (table 2). Compensation will be provided to participants at each follow-up time point to improve retention. At each time point, participants will report if they experienced PFP in the previous 6 months and the average hours per week engaged in running or exercise in the previous 4 weeks.^17^ Recurrence will be defined as an event that requires treatment from a healthcare professional or being unable to participate in normal exercise for more than 2 days due to retropatellar or peripatellar pain.^17^ If additional clarification is required, follow-up will be completed with the research team member and participant through email.

### Secondary outcomes

Hip muscle function will be assessed with peak torque and RTD during isometric contractions for abduction and extension with a hand-held dynamometer (HHD) (ErgoFet HHD, Hoggan Scientific, Salt Lake City, Utah, USA). For each task, a participant will be positioned with the HHD and taken through a series of sub-maximal isometric contractions to allow for warm-up and acclimation. The warm-up will consist of contractions at 25, 50, 75 and 100% of their self-perceived maximal isometric contraction. Muscle function will consist of three 5 s maximal contractions with verbal encouragement. Participants will be provided with a 1 min rest period between trials and 3 min rest period between testing positions, with positions completed in a randomised order varied over sessions. We will collect an additional test trial if coefficient of variation is greater than 10% between the three peak isometric torque trials.^34^ We calculated inter-rater reliability of gluteal RTD (inter-rater ICC=0.66−0.78) and isometric hip strength (inter-rated ICC=0.83−0.88; intrarater ICC: 0.80−0.98) between the three site assessors during a training session. Hip muscle function will be collected preintervention and immediately postintervention.

The dynamometer will be calibrated before each testing session. Thigh and shank length will be measured in centimetres for the test limb. Hip abduction will be conducted in a side-lying position with the test limb positioned at 20° of hip abduction, slight external rotation and slight hip extension.^19^ The HHD will be placed on the lateral aspect of the limb, 5 cm proximal to the lateral epicondyle and secured with a stabilisation strap.^19^ A second stabilisation strap will be placed around the table and participant’s pelvis to minimise accessory motion. Hip extension will be conducted with the participant’s trunk on the table and their lower limbs off the table, with both the hip and knee at 90° of flexion. The HHD will be positioned against the treatment plinth and a stabilisation strap placed around the HHD and participant’s limb, aligned 5 cm proximal to the popliteal fossa. A second stabilisation trap will be positioned around the table and the participant’s pelvis to control accessory motion.

Frontal plane kinematics will be assessed during a single leg squat (SLS). Participants will wear tight-fitting clothes without shoes for the two-dimensional (2D) SLS assessment. Participants will complete an SLS on the pathological limb, with their contralateral limb flexed to 90° and arms placed across their chest.^35^ Participants will squat as low as possible and return to starting position.^35^ Coloured stickers will be placed on anatomical landmarks (sternum, bilateral anterior superior iliac spine (ASIS) and patella) to help quantify frontal knee, hip, pelvis and trunk kinematics. Video instructions will be provided to participants for sticker placement, camera placement and standardised squatting instructions. Squat speed will be standardised to a 2 s descend and 2 s ascend.^35^ Three practice trials will be provided before data collection, followed by 1 min rest, then three recorded trials. Researchers will assess and provide feedback on marker placement, camera placement and squatting performance at both baseline and immediate postintervention prior to data collection to improve accuracy. The feedback will serve as training for participants who will collect squatting performance remotely at the 6, 12, 18 and 24 months time points.

We will collect a global rating of change (GROC) score to quantify patient perceived change at all postintervention time points (immediate, 6- 12, 18 and 24 months). The GROC uses a single 7-point Likert-type scale with anchors of very much improved to very much worse. Also, each participant will be asked at the 6, 12, 18 and 24 months time points if they received additional care or treatment in the previous 6 months for their PFP. Those who have received care will be asked additional questions related to the type, reason and duration of care.

### Data management

Force data will be normalised to body mass and converted to torque (Nm/kg). The mean of the three test trials will determine the peak isometric torque. RTD for each muscle will be calculated by the change in torque divided by a specific duration during the early (0–50 ms) and late (100–200 ms) phases. The RTD will be normalised to body mass and reported as Nm/kg/s. A custom MATLAB code will calculate RTD.^19^

We will measure frontal plane kinematics for the knee, hip, pelvis and trunk at both single leg stance and peak knee flexion of the SLS. Knee kinematics (knee-frontal plane projection angle (FPPA)) will be defined as the angle with bisecting lines between the ankle mortise, centre of the patella and the ASIS.^35^ Hip kinematics (hip-FPPA) will be defined as the angle between the centre of the patella, ipsilateral ASIS and contralateral ASIS.^35^ Pelvic drop will be defined as the angle between the horizontal line (aligned with the testing location), ipsilateral ASIS and contralateral ASIS. Trunk kinematics (lateral trunk motion) will be measured with the angle between a vertical line (aligned with the testing location), ipsilateral ASIS and manubrium sternum. There is good to excellent between session reliability (ICC=0.70−0.91) and intrarater (ICC _(2,1)_ = 0.75−0.90) in females with PFP.^35^ A single research team member, who will be blinded to group allocation, will measure the average of the three trials for all data points.

### Sample size

Power analysis for preintervention to postintervention differences was conducted with a Monte Carlo simulation involving 5000 replications of the data via the interval Monte Carlo simulation capabilities of Mplus V.8.6 for the multivariate analysis of covariance (MANCOVA) assuming: (1) standardisation of all analysis variables, (2) randomisation results in equivalent groups across the outcome variables at the baseline assessment, (3) homogeneity of covariate regression slopes (ie, 6-week postintervention outcome variables regressed onto their respective baseline assessment counterparts) will be ensured using parameter estimate equality constraints, (4) missing completely at random (MCAR) or missing at random (MAR) missing data were included in the simulation and ranged between 4.5% and 15.9% pairwise missing data (average=10%), (5) all six outcome variables were assumed to be intercorrelated at a medium effect size (ie, *r*_Pearson_=0.50), (6) baseline assessment scores for all six outcome variables, included as covariates, will explain (*R*^2^=0.20) 20% of 6-week postintervention variance, (7) maximum likelihood missing data handling will make N=88 available for analysis and (8) α=0.05 (two tailed). The power analysis question of interest was the smallest *d* incremental difference (*d*) at 6-week postintervention for STRIPE versus SOC participants detectable with power at 0.80. Power analysis results showed power would be >0.80 for any effect size difference between STRIPE and SOC participants of d>0.55.

Power analysis for the four postintervention (6, 12, 18 and 24 months) assessments was calculated with a Monte Carlo simulation involving 5000 replications of sample data using Mplus V.8.6 for the intercept-only structural equation model (SEM; growth curve model)^36^ assuming: (1) standardisation of all outcome variables at all time points, (2) *T*=five 6-month assessment time points (postintervention, 6, 12, 18 and 24 months follow-up), (3) all individual trajectories are horizontal but at different (intercept) levels, (4) randomisation results in equivalent groups, (5) intermittent missing outcome variable data will be MAR, increase every 6 months and monotonically from 1.1% at the first postfollow-up assessment to 9.1% at month 24 and (6) α=0.05 (two tailed). The dispositive power analysis question regarded statistical power levels to reject the treatment effect null hypothesis at various hypothetical sample sizes under the assumptions listed above. Power analysis results showed power would be >0.70 for any (intercept) effect size difference between STRIPE and SOC participants of d>0.55.

Power analysis for PFP recurrence was assessed with a binary logistic regression using the internal Monte Carlo simulation capabilities in Mplus V.8.6 assuming: (1) 50% of the STRIPE participants will have a PFP recurrence and 82% of SOC participants will have recurrence, (2) previous publications suggest PFP recurrence for SOC is 50%–90%,^6^ (3) MCAR missing data were included in the simulation and estimated to be 20% missing data in the outcome variable (PFP recurrence) and (4) α=0.05 (two tailed). Power analysis results showed power >0.80 if N=88 for analysis following missing data handling and the group (STRIPE vs SOC) term results in an OR>4.43.

### Statistical analyses

We will use a MANCOVA to test for a significant treatment group (STRIPE vs SOC) main effect showing significantly greater improvement in each outcome variable at 6-week postintervention controlling for respective baseline values of each outcome variable. A multivariate intercept-only longitudinal SEM growth curve model will be used to determine if the difference in each outcome variable at 6-week postintervention is maintained at 6, 12, 18 and 24 months follow-up. The intercept-only SEM implies between-participant variability in the overall outcome variable, but the outcome does not change as a function of time. Finally, a binary logistic regression analysis will be used to determine if the intervention group (STRIPE vs SOC) significantly predicts PFP recurrence at the end of the 2-year follow-up. Dummy coded variables will be created and added to each analysis to account for cluster (site) differences and produce robust SEs. All missing data will be handled assuming MAR with maximum likelihood estimation consistent with currently accepted methodological research and practice.

## Discussion

This protocol outlines the methodology for a RCT comparing SOC and STRIPE interventions on pain, subjective function, recurrence rates and muscle function in individuals with PFP. While there is preliminary evidence that power-based rehabilitation is effective for PFP, this is the first study to evaluate the effectiveness of PFP recurrence or hip muscle function from a neuromuscular and biomechanical perspective. The findings of this study may inform clinicians who commonly treat PFP to prescribe power-based exercises to improve short-term and long-term outcomes.

### Ethics and dissemination

This study has been approved by the University of Connecticut Institutional Review Board (IRB# HR22-0038) and approved by the Department of Defense’s Human Research Protection Official. Participants will be informed of any amendments to the protocol, with corresponding updates to ClinicalTrials.gov. Study results will be disseminated through peer-reviewed publications in discipline-specific journals, conference presentations and to the Department of Defense. Authorship will be provided to individuals who contribute to study design, data collection and data analysis.

### Data monitoring and adverse events

Due to the minimal risk in the intervention, this study will not have a data monitoring committee or interim analysis to terminate the study. All investigators and research team members will complete training before participant enrolment. Training will include reviewing classification of adverse events and evaluation of relatedness of adverse events. All adverse events will be reported to the PI, who will submit documentation to the University of Connecticut Institutional Review Board. The site investigators will meet every 3 months to maintain an appropriate response for adverse events and the required course of action. There is minimal risk for injury for those participants who enrol in the study, as the intervention delivers exercises prescribed in clinical practice; however, no ancillary/post-trial care or compensation will be provided to participants who suffer harm from the trial. Any adverse events will be reported to the study PI within 5 days of their knowledge of the event for submission to the institutional review board.

### Limitation

This study does have some limitations. Due to the heterogeneous presentation of impairments in individuals with PFP, the magnitude of neuromuscular deficits is unknown. Cross-sectional evidence does support that RTD of the gluteal muscles exists in individuals with PFP compared with asymptomatic individuals supporting our intervention. Another limitation is that the study is only comparing two neuromuscular-focused interventions. Biomechanical impairments may be treated with movement retraining; however, neuromuscular programmes are recommended to include exercises for the gluteal and quadriceps muscles. Participants will collect SLS kinematics remotely at the final four time points, stressing the importance of accurate marker and camera placement. The researchers will train and assess participant’s ability to collect this data at both the baseline and immediate postintervention to improve the future remote assessments. Additionally, we purposely selected to the patella as a marker location instead of midpoint of medial and lateral epicondyles, as the patella has been used in previous literature to calculate 2D kinematics^35^ and to improve participant ability to identify the landmark for marker placement. Finally, our intervention is a 6-week programme, but the optimal dosage for treating PFP is unknown. Preliminary data have used a longer intervention duration; adherence was poor around the sixth week, supporting our intervention length.
