# Supplementary material for: Strength Training Rehabilitation Incorporating Power Exercises (STRIPE) for individuals with patellofemoral pain: a randomised controlled trial protocol
Source: BMJ Open Sport Exerc Med. 2023 Jan 17;9(1):e001482. doi: 10.1136/bmjsem-2022-001482 (PMC9853263; doi:10.1136/bmjsem-2022-001482)
Supplement: Supplementary data [file bmjsem-2022-001482supp001.pdf]

Supplementary File 1: SPIRIT Checklist

SPIRIT

STANDARD PROTOCOL ITEMS: RECOMMENDATIONS FOR INTERVENTIONAL TRIALS

SPIRIT 2013 Checklist: Recommended items to address in a clinical trial protocol and related documents*

Section/item	Item No	Description	Addressed on page number
**Administrative information**			
Title	1	Descriptive title identifying the study design, population, interventions, and, if applicable, trial acronym	Page 1
Trial registration	2a	Trial identifier and registry name. If not yet registered, name of intended registry	Page 3/7
	2b	All items from the World Health Organization Trial Registration Data Set	Page 3/7
Protocol version	3	Date and version identifier	Page 1
Funding	4	Sources and types of financial, material, and other support	Page 1
Roles and responsibilities	5a	Names, affiliations, and roles of protocol contributors	Page 1
	5b	Name and contact information for the trial sponsor	Page 1
	5c	Role of study sponsor and funders, if any, in study design; collection, management, analysis, and interpretation of data; writing of the report; and the decision to submit the report for publication, including whether they will have ultimate authority over any of these activities	Page 1

	5d	Composition, roles, and responsibilities of the coordinating centre, steering committee, endpoint adjudication committee, data management team, and other individuals or groups overseeing the trial, if applicable (see Item 21a for data monitoring committee)	Page 1
**Introduction**			
Background and rationale	6a	Description of research question and justification for undertaking the trial, including summary of relevant studies (published and unpublished) examining benefits and harms for each intervention	Page 5-7
	6b	Explanation for choice of comparators	Page 6-7
Objectives	7	Specific objectives or hypotheses	Pages 6-7
Trial design	8	Description of trial design including type of trial (eg, parallel group, crossover, factorial, single group), allocation ratio, and framework (eg, superiority, equivalence, noninferiority, exploratory)	Page 8
**Methods: Participants, interventions, and outcomes**			
Study setting	9	Description of study settings (eg, community clinic, academic hospital) and list of countries where data will be collected. Reference to where list of study sites can be obtained	Page 8
Eligibility criteria	10	Inclusion and exclusion criteria for participants. If applicable, eligibility criteria for study centres and individuals who will perform the interventions (eg, surgeons, psychotherapists)	Page 8-9, Table 1
Interventions	11a	Interventions for each group with sufficient detail to allow replication, including how and when they will be administered	Page 9-11, Table 2 Supp 2-4

	11b	Criteria for discontinuing or modifying allocated interventions for a given trial participant (eg, drug dose change in response to harms, participant request, or improving/worsening disease)	Page 8
	11c	Strategies to improve adherence to intervention protocols, and any procedures for monitoring adherence (eg, drug tablet return, laboratory tests)	Page 10-11
	11d	Relevant concomitant care and interventions that are permitted or prohibited during the trial	Page 12, 13 and 15
Outcomes	12	Primary, secondary, and other outcomes, including the specific measurement variable (eg, systolic blood pressure), analysis metric (eg, change from baseline, final value, time to event), method of aggregation (eg, median, proportion), and time point for each outcome. Explanation of the clinical relevance of chosen efficacy and harm outcomes is strongly recommended	Page 11-14
Participant timeline	13	Time schedule of enrolment, interventions (including any run-ins and washouts), assessments, and visits for participants. A schematic diagram is highly recommended (see Figure)	Page 11-12 and Table 3
Sample size	14	Estimated number of participants needed to achieve study objectives and how it was determined, including clinical and statistical assumptions supporting any sample size calculations	Page 15-17
Recruitment	15	Strategies for achieving adequate participant enrolment to reach target sample size	Page 8

Methods: Assignment of interventions (for controlled trials)

Allocation:

Sequence generation	16a	Method of generating the allocation sequence (eg, computer-generated random numbers), and list of any factors for stratification. To reduce predictability of a random sequence, details of any planned restriction (eg, blocking) should be provided in a separate document that is unavailable to those who enrol participants or assign interventions	Page 8-9
Allocation concealment mechanism	16b	Mechanism of implementing the allocation sequence (eg, central telephone; sequentially numbered, opaque, sealed envelopes), describing any steps to conceal the sequence until interventions are assigned	Page 8-9
Implementation	16c	Who will generate the allocation sequence, who will enrol participants, and who will assign participants to interventions	Page 8-9
Blinding (masking)	17a	Who will be blinded after assignment to interventions (eg, trial participants, care providers, outcome assessors, data analysts), and how	Page 8-9
	17b	If blinded, circumstances under which unblinding is permissible, and procedure for revealing a participant's allocated intervention during the trial	Page 8-9

Methods: Data collection, management, and analysis

Data collection methods	18a	Plans for assessment and collection of outcome, baseline, and other trial data, including any related processes to promote data quality (eg, duplicate measurements, training of assessors) and a description of study instruments (eg, questionnaires, laboratory tests) along with their reliability and validity, if known. Reference to where data collection forms can be found, if not in the protocol	Page 9 and 18
	18b	Plans to promote participant retention and complete follow-up, including list of any outcome data to be collected for participants who discontinue or deviate from intervention protocols	Page 13

Data management	19	Plans for data entry, coding, security, and storage, including any related processes to promote data quality (eg, double data entry; range checks for data values). Reference to where details of data management procedures can be found, if not in the protocol	Page 14 and 15
Statistical methods	20a	Statistical methods for analysing primary and secondary outcomes. Reference to where other details of the statistical analysis plan can be found, if not in the protocol	Page 17
	20b	Methods for any additional analyses (eg, subgroup and adjusted analyses)	N/A
	20c	Definition of analysis population relating to protocol non-adherence (eg, as randomised analysis), and any statistical methods to handle missing data (eg, multiple imputation)	Page 17
**Methods: Monitoring**			
Data monitoring	21a	Composition of data monitoring committee (DMC); summary of its role and reporting structure; statement of whether it is independent from the sponsor and competing interests; and reference to where further details about its charter can be found, if not in the protocol. Alternatively, an explanation of why a DMC is not needed	Page 18-19
	21b	Description of any interim analyses and stopping guidelines, including who will have access to these interim results and make the final decision to terminate the trial	Page 18-19
Harms	22	Plans for collecting, assessing, reporting, and managing solicited and spontaneously reported adverse events and other unintended effects of trial interventions or trial conduct	Page 18-19
Auditing	23	Frequency and procedures for auditing trial conduct, if any, and whether the process will be independent from investigators and the sponsor	Page 18-19
**Ethics and dissemination**			

Research ethics approval	24	Plans for seeking research ethics committee/institutional review board (REC/IRB) approval	Page 18
Protocol amendments	25	Plans for communicating important protocol modifications (eg, changes to eligibility criteria, outcomes, analyses) to relevant parties (eg, investigators, REC/IRBs, trial participants, trial registries, journals, regulators)	Page 18
Consent or assent	26a	Who will obtain informed consent or assent from potential trial participants or authorised surrogates, and how (see Item 32)	Page 9
	26b	Additional consent provisions for collection and use of participant data and biological specimens in ancillary studies, if applicable	N/A
Confidentiality	27	How personal information about potential and enrolled participants will be collected, shared, and maintained in order to protect confidentiality before, during, and after the trial	Page 18-19
Declaration of interests	28	Financial and other competing interests for principal investigators for the overall trial and each study site	Page 1
Access to data	29	Statement of who will have access to the final trial dataset, and disclosure of contractual agreements that limit such access for investigators	Page 19
Ancillary and post-trial care	30	Provisions, if any, for ancillary and post-trial care, and for compensation to those who suffer harm from trial participation	Page 19
Dissemination policy	31a	Plans for investigators and sponsor to communicate trial results to participants, healthcare professionals, the public, and other relevant groups (eg, via publication, reporting in results databases, or other data sharing arrangements), including any publication restrictions	Page 18
	31b	Authorship eligibility guidelines and any intended use of professional writers	Page 18

	31c	Plans, if any, for granting public access to the full protocol, participant-level dataset, and statistical code	Page 19
**Appendices**			
Informed consent materials	32	Model consent form and other related documentation given to participants and authorised surrogates	N/A
Biological specimens	33	Plans for collection, laboratory evaluation, and storage of biological specimens for genetic or molecular analysis in the current trial and for future use in ancillary studies, if applicable	N/A

*It is strongly recommended that this checklist be read in conjunction with the SPIRIT 2013 Explanation & Elaboration for important clarification on the items. Amendments to the protocol should be tracked and dated. The SPIRIT checklist is copyrighted by the SPIRIT Group under the Creative Commons "Attribution-NonCommercial-NoDerivs 3.0 Unported" license.

Supplementary File 2: TIDieR-Telehealth checklist

Set of Descriptors	Location
BRIEF NAME<br>1. Provide the name or a phrase that describes the intervention	Pages 9-11
WHY<br>2. Describe any rationale, theory, or goal of the elements essential to the intervention	Pages 9-11
WHAT<br>3. Materials: Describe any physical or informational materials used in the intervention, including those provided to participants or used in intervention delivery or in the training of intervention providers. Provide information on where the materials can be accessed (e.g. online appendix, URL).	Pages 9-11
4. Procedures: Describe each of the procedures, activities, and/or processes used in the intervention, including any enabling or support activities.	Pages 9-11
WHO PROVIDED<br>5. For each category of intervention provider (e.g. psychologist, nursing assistant), describe their expertise, background and any specific training given.	Pages 10-11
HOW<br>6. Describe the modes of delivery (e.g., face-to-face or by some other mechanism, such as internet or telephone) of the intervention and whether it was provided individually or in a group.	Pages 10-11
WHERE<br>7. Describe the type(s) of location(s) where the intervention occurred, including any necessary infrastructure or relevant features.	Page 10
WHEN AND HOW MUCH<br>8. Describe the number of times the intervention was delivered and over what period of time including the number of sessions, their schedule, and their duration, intensity or dose.	Pages 9-10
TAILORING<br>9. If the intervention was planned to be personalized, titrated or adapted, then describe what, why, when, and how.	Pages 10-11
MODIFICATIONS<br>10. If the intervention was modified during the course of the study, describe the changes (what, why, when, and how).	N/A
HOW WELL<br>11. Planned: If intervention adherence or fidelity was assessed, describe how and by whom, and if any strategies were used to maintain or improve fidelity, describe them.	Pages 10-11
12. Actual: If intervention adherence or fidelity was assessed, describe the extent to which the intervention was delivered as planned.	N/A

Supplementary File 3: Consensus on Exercise Reporting Template Guidelines Checklist

**Set of Descriptors**	**Location**
Type of exercise equipment	Page 10
Qualifications, teaching/supervision expertise, and/or training of exercise instructors	Page 10-11
Whether exercises are performed individually or in a group	Page 10
Whether exercise are supervised or unsupervised	Page 10
Measurement and reporting of adherence to exercise	Page 11
Details of motivation strategies	Page 11
Decision rules for progression the exercise program	Page 11
Each exercise is described so that it can be replicated	Figure 1
Content of any home program component	Pages 10-11
Nonexercise components	Page 10
How adverse events that occur during exercise are documented and managed	Page 11/18
Setting in which exercises are performed	Page 10
Detailed description of the exercises	Table 2
Whether exercises are generic (“one size fits all”) or tailors to the individual	Pages 10-11
Decision rule that determines the starting level for exercise	Page 10
Whether the exercise intervention is delivered and performed as planned	N/A

Supplementary File 4: Toigo and Boutiller Checklist

Set of Descriptors	Location
Load Management	Table 2
Number of Repetitions	Table 2
Number of Sets	Table 2
Rest in-between sets	Table 2
Number of exercise interventions	Pages 9-10
Duration of experiment	Pages 9-10
Fractional and temporal distribution of contraction	Table 2
Rest in-between repetitions	Table 2
Time under tension	Table 2
Volitional muscular failure	Page 10
Range of Motion	Figure 1
Recovery time in-between exercise sessions	Table 2
Anatomical definition of exercises	Figure 1

Supplementary File 5: REPORT-PFP Checklist

REPORT-PFP CHECKLIST 2021

Checklist of strongly recommended and recommended items for quantitative patellofemoral pain studies

Section 1 – Items Strongly Recommended (Essential)		Reported on page # or N/A
Demographics		
1	Sex or gender of the participants	Page 11
2	Age of the participants	Page 11
Baseline symptoms		
3	Symptom duration	Page 11
4	Pain Severity	Page 11
5	Unilateral/bilateral symptoms	Page 11
Outcome measures		
6	Condition specific patient-reported outcome	Page 12
7	Pain severity	Page 12
Outcome measure description		
8	Describe assessment in adequate detail to allow replication	Pages 8-14
Reporting study results		
9	Mean and standard deviation for parametric data	N/A
10	Median and interquartile range for non-parametric data	N/A
11	Precision of estimate for all inferential statistics (e.g. 95% confidence interval for between group differences)	N/A
**Section 2 – Items Recommended (encouraged but are not required to meet consensus recommendations)**		**Reported on page # or N/A**
Demographics		
12	Anthropometrics (including body mass and height or body mass index)	Page 11
13	Physical activity levels	Page 9
14	Source/setting/location of participants	Page 8
15	Ethnicity of the participants	Page 11

Baseline symptoms and previous treatment		
16	Previous treatment	Page 11
17	Pain location(s)	Page 11
18	Aggravating factors	Page 11
19	History of knee surgery	Page 11
20	Other symptoms, musculoskeletal symptoms, and comorbidities	Page 11
21	Crepitus	Page 11
22	Pain quality	Page 11
Outcome measures		
23	Physical activity	Page 9
24	Global rating of change	Page 14
25	Health-related quality of life	Page 9
26	Psychological factors (including self-efficacy, pain-related fear and pain catastrophising)	Page 9
Outcome measure description		
27	Provide measurement properties of assessments	Pages 12-14
28	Provide videos and/or images of assessments	Figure 1
Clinical trial methodology		
29	Follow recommendations from EQUATOR Network[2]	N/A (Spirit – Supp File 1)
30	Use existing checklists for interventions, including TIDiER; CERT for exercise interventions; and Toigo and Boutellier for resistance training interventions	Supplementary Files 2-4
31	Provide videos and/or images of treatments	Figure 1

N/A = not applicable

CERT = Complete Exercise Reporting Template[1]; EQUATOR = Enhancing the QUAlity and Transparency Of health Research[2]; TIDiER = Template of Intervention Description and Replication[3].
